# Spray-Dried Salt-Starch Particles for Sodium Reduction and Saltiness Maintenance in Extruded Snacks

**DOI:** 10.3390/foods15050804

**Published:** 2026-02-25

**Authors:** Crislayne Teodoro Vasques, Ana Clara Souza, Any Caroliny Santos de Arcantes, Gabriel Sarache, Bruno Henrique Figueiredo Saqueti, Carlos Eduardo Barão, Oscar Oliveira Santos, Tatiana Colombo Pimentel, Antonio Roberto Giriboni Monteiro

**Affiliations:** 1 Postgraduate Program in Food Science, State University of Maringá (UEM), Maringa 87020-900, Brazilgabrielsarache481@gmail.com (G.S.); oosjunior@uem.br (O.O.S.J.); argmonteiro@uem.br (A.R.G.M.); 2 Postgraduate Program in Food Engineering, State University of Maringá (UEM), Maringa 87020-900, Brazil; anacsouza00@gmail.com; 3 Department of Food Engineering, State University of Maringá (UEM), Maringa 87020-900, Brazil; ra123850@uem.br; 4 Department of Chemistry, State University of Maringá (UEM), Maringa 87020-900, Brazil; bruno_saqueti@outlook.com; 5 Federal Institute of Paraná (IFPR), Paranavaí Campus, Paranavaí 87703-536, Brazil; carlos.barao@ifpr.edu.br

**Keywords:** sodium reduction, saltiness perception, snacks, extrusion process, corn grits

## Abstract

Excessive sodium consumption is a global public health problem that demands technological innovations in processed foods. This study aimed to reduce the sodium content in extruded corn snacks while maintaining perceived saltiness by substituting common salt with compound microparticles (70% NaCl, 30% starch). Two drying methods were evaluated: spray drying and conventional oven drying. The snacks were subjected to physicochemical, instrumental (texture and colour), density, porosity, microstructural, and sensory analyses (intensity scale, n = 104). The results demonstrated that the particles obtained by spray drying allowed a 28% reduction in the final sodium content without statistically differing in saltiness perception compared to the control. In contrast, the oven treatment reduced saltiness perception compared to the standard. Images obtained by scanning electron microscopy, along with porosity measurements, demonstrated a significant increase in porosity in the spray-dried sample. This allows rapid dissolution of the salt in the mouth, maintaining a salty taste even with reduced sodium content. It was concluded that the use of salt–starch microparticles via spray drying was a viable strategy for producing snacks with reduced sodium content without compromising sensory quality.

## 1. Introduction

The World Health Organisation (WHO) estimates that 15 million people aged 30 to 69 die prematurely due to noncommunicable chronic diseases (NCDs). These diseases can affect people of all ages, ethnicities, and cultures. In recent years, even children have become vulnerable to the risk factors that contribute to their development. Risk factors for NCDs include inadequate dietary patterns, physical inactivity, smoking, and excessive alcohol consumption [1].

Inadequate sodium intake has raised significant concern for the World Health Organisation (WHO), as the global population’s consumption of this substance far exceeds established recommendations. For adults, the WHO recommends a maximum intake of 2 g of sodium per day, equivalent to 5 g of salt. However, most people around the world consume between 3 and 6 g of sodium daily, and fewer than 5–10% ingest less than 2.3 g/day [2,3]. Robust scientific reviews corroborate the harmful effects of excess sodium on the cardiovascular system [4], unequivocally recommending salt reduction as one of the most effective strategies to decrease cardiovascular diseases. However, implementing this reduction remains challenging, especially given the fundamental role of salt in the palatability of ready-to-eat foods [5].

In this context, extruded snacks stand out for their high sodium content, posing a significant nutritional concern. Although this type of product enables high process standardisation and economic feasibility with raw materials such as corn semolina, there remains a need to develop and implement strategies to improve its nutritional profile, particularly by reducing sodium, without compromising sensory characteristics [6,7,8].

Extrusion technology consists of a process in which a material is subjected to high temperatures and pressures and forced through a die or opening with the desired cross-section [7,8], promoting the rapid cooking of cereals and causing slight changes in their nutritional characteristics. This technology enables the production of foods with lower processing costs, continuous operation, high yields, and better quality, with optimised energy use [6].

Various raw materials can serve as bases for extruded products, including corn. In the food industry, this cereal must be processed by reducing grain size through milling steps. The previously dried grain undergoes successive phases of grinding and sieving, resulting in particles of different sizes that determine the type of product obtained, such as cornmeal and grits, which are the primary raw materials used in snack production [6]. Wani and Kumar [9], in their investigation into the enrichment of extruded products with nutritious and healthy ingredients, highlighted that their physical and functional properties remain underexplored and could be further enhanced to enhance their nutritional value.

In processed foods, the distribution of salt within the food matrix plays a crucial role in saltiness perception. It may be explored as an alternative to reduce sodium content without compromising sensory characteristics [10,11,12]. Salt perception in solid foods is not only determined by the total amount of salt present but by the release kinetics of this compound and the concentration reached at the gustatory receptors on the tongue [8].

Studies on baked products, such as bread, have shown that creating a nonhomogeneous salt distribution, mainly by concentrating it on the surface, significantly increases salt perception without raising total sodium content [13]. This phenomenon is attributed to the rapid initial dissolution of salt crystals during mastication, resulting in a high, transient salt concentration near the surface. In extruded matrices, this principle is highly relevant because seasonings are usually applied superficially after extrusion. However, incorporating salt into the dough before extrusion traps it within the gelatinised starch matrix, leading to a slower release and, consequently, lower perceived saltiness at the same concentration [14].

Given that salt plays an essential role in the palatability of extruded snacks, adopting technological strategies to reduce its use is particularly relevant. In this context, particle-engineering approaches applied during the coating stage have emerged as promising alternatives, enabling modulation of sodium release without altering the extruded matrix. Gao et al. [15] emphasise that consumers tend to react negatively to unexpected formulation changes, making it essential that any sensory modifications be compensated for through appropriate visual or nutritional information.

The incorporation of compounds into carrier systems represents a practical approach for developing foods with improved quality, as it enables modulation of ingredient distribution and release behaviour. Starch, being a biopolymer that is widely available in nature, stands out as a promising carrier and structuring material due to its low cost, high availability, and functional versatility, including its high-water retention capacity and ability to modulate viscosity. Moreover, starch is an essential food ingredient and, when subjected to physicochemical modifications, can exhibit excellent film-forming, emulsifying, and resistant properties, which are highly desirable in carrier and structuring systems for controlled release applications [16].

Based on this reasoning, the working hypothesis of the present study is that modifying the structure of salt particles through starch-based composite systems can alter the perception of sodium on the tongue, thereby maintaining salinity perception with a lower sodium load.

Accordingly, the present study aims to evaluate the effect of salt–corn starch composite particles obtained via different drying methods on saltiness perception in an extruded snack.

## 2. Materials and Methods

Initially, a pre-test was conducted using the spray dryer solely to determine the amount of salt that could be reduced. In these tests, sodium was replaced with spray-dried starch at 10%, 30%, and 50% of the concentration. No reduction in perceived salinity was observed at the 10% and 30% reductions relative to the standard, but tasters reported a significant decrease at the 50% reduction. Then, to evaluate the efficiency of salt–starch structuring and the effect of the drying method on the properties of the final product, three different treatments were applied: (SD) spray-dried composite microparticles of salt and corn starch; (OD) mixture of salt and starch dried in a conventional oven; and (C) pure salt, used as a control.

The oven-dried sample (OD), with the same salt-to-starch ratio as the spray-dried sample (SD), was used as a process control to isolate the effect of the drying method from that of the formulation (simple dilution). Thus, differences between SD and OD can be attributed to the drying process rather than to composition.

### 2.1. Obtaining the Sprayed Salt

To produce the sprayed salt, a solution containing 70 g of salt and 30 g of corn starch dispersed in 900 g of water was prepared. The mixture was homogenised using a magnetic stirrer for 5 min at room temperature. Subsequently, the dispersion was dried in a benchtop spray dryer at a feed rate of 3.6 L/h and an air outlet temperature of 105 °C (LabMaq/SD 5.0/Brazil). The process was conducted with an inlet temperature at the spray nozzle of approximately 165 °C, maintaining the feed until complete atomisation and sample drying.

### 2.2. Obtaining Salt with Starch by Oven Drying

For the oven treatment, the same solids-to-solvent ratio as in the previous method was used (70 g of salt, 30 g of starch, and 900 g of water). Unlike the last process, the mixture was kept under magnetic stirring at 75 °C until complete gelatinisation of the starch. The gelatinised mixture was poured into wide trays to form a thin layer (approximately 2 cm thick) and then dried in a forced-air oven at 55 °C for 24 h, yielding dry plates. The dried material was then ground in a knife mill to obtain a fine, uniform powder (20–40 µm).

### 2.3. Development of Raw Materials, Extrusion Processes, and Formulations

To ensure standardised particle size across all salt treatments, all samples (SD, OD, and C) were sieved to 20–40 µm, discarding particles outside this size range. To guarantee standardised moisture content, all samples were kept in a desiccator for 48 h before use.

The snacks were produced from corn kernels supplied by Nutrimilho Alimentos (Maringá, Paraná, Brazil). For the coating, delicate herb seasoning (All-Flavours, Diadema, SP, Brazil), sodium chloride (Moc, Mossoró, RN, Brazil), and corn oil (Cocamar, Maringá, Paraná, Brazil) were used. Before extrusion, 2.5% water was added to the kernels, stabilising the moisture content at approximately 16% *w*/*w*. The mixture was homogenised for 10 min in a vertical mixer at 60 rpm to ensure uniform moisture distribution.

Extrusion was carried out using a single-screw extruder (RX 50, Imbramaq, Ribeirão Preto, SP, Brazil) with a 3.0 mm die. Operational parameters were kept constant for all samples: screw speed of 150 rpm, die zone temperature of 120 °C, and feed rate of 30 g/s. After extrusion, the snacks were dried in a forced-air oven for 60 min and stored in polyethene bags until the flavouring stage.

During the flavouring stage, three salt treatments were applied to the snacks: 880 g of extruded products were placed in a rotating drum, 100 mL of corn oil was sprayed using a compressor (Schulz/CSD9, Joinville, SC, Brazil), and 20 g of each salt sample was manually applied. The treatments were: (SD) spray-dried composite microparticles composed of salt and corn starch (70/30 *w*/*w*); (OD) mixture of salt and starch dried in an oven (70/30 *w*/*w*); and (C) pure salt, used as a control (100%).

### 2.4. Determination of Centesimal Composition, Water Activity (aw), and Sodium Content

Moisture (oven-drying at 105 °C), ash (600 °C), protein (Kjeldahl), lipids (Soxhlet), and carbohydrate content were determined by AOAC methods [17]. The water activity (aw) of the samples was measured at 25 °C using the AQUALAB (Dew Point Water Activity Metre 4te, Pullman, WA, USA). The sodium content of the samples was quantified by flame atomic absorption spectrophotometry, using the A 300 model equipment (Analytik Jena A6, Jena, Germany).

### 2.5. Determination of the Expansion Rate, Specific Volume, Retraction Index, and Hardness

The expansion rate (ER) and specific volume (SV) were determined as described by Genç and Nizamlıoğlu [18]. The ER was calculated as the ratio of the average diameter of 10 extruded snacks to the extruder die diameter, using a digital micrometre with a resolution of 0.001 mm (Mitutoyo, Hiroshima, Japan). The SV was calculated as the ratio of the average mass of the 10 snacks to their average volume. The retraction index (*RI*) was determined as the negative change in specific volume during storage, calculated after 7 days using Equation (1).

(1) $$RI=\frac{final\;specific\;volume}{initial\;specific\;volume}\;$$

The instrumental hardness of the snacks was determined using a TAXT2 Plus texture analyser (Stable Micro Systems, Godalming, UK) [18].

### 2.6. Determination of Colour

The colour of the samples was analysed using a CR-400 colourimeter (Konica Minolta, Tokio, Japan), with three readings per treatment. The results were expressed in the CIELAB system, using the luminance (L*) and chromatic coordinates a* and b* [19].

### 2.7. Density and Porosity

Loose bulk density (LBD), packed bulk density (PBD), and true density (TD) were determined by the procedure of Falade et al. (2019) [20]. Packed bulk density (PBD) was determined after tapping the sample until no change in volume was observed. True density (TD) was determined by the displacement method. About 2 g of sample was weighed in a 10 mL measuring cylinder containing n-hexane at the 5 mL mark in less than 10 s to reduce swelling, adsorption, and volatility. The density values were calculated using Equations (2)–(4) and expressed in g/mL. Porosity (%) was determined as shown in Equation (5) and expressed in %.

(2) $$Loose\;bulk\;density\;(g\;{mL}^{-1})=\frac{bulk\;mass\;of\;sample}{bulk\;volume}\;$$

(3) $$Packed\;bulk\;density\;(g\;{mL}^{-1})=\frac{mass\;of\;tapped\;sample}{tapped\;volume}\;$$

(4) $$True\;density\;(g\;{mL}^{-1})=\frac{mass\;of\;sample}{displaced\;volume}$$

(5) $$Pososity\;\left(\%\right)=100\left(1-\frac{bulk\;density}{true\;density}\right)$$

### 2.8. Scanning Electron Microscopy (SEM)

The physical and structural characteristics of the salt–starch particles were analysed by scanning electron microscopy (SEM) using a Quanta 250 ESEM (FEI). Samples were mounted on 20 mm diameter cylindrical metal stubs using conductive double-sided carbon adhesive tape and were sputter-coated with gold prior to imaging. Micrographs were acquired at accelerating voltages of 5 and 10 kV using an Everhart–Thornley detector (ETD), with a spot size setting of 3. Images were collected at magnifications of 1000× and 5000×, with scale bars of 100 µm and 30 µm, respectively.

### 2.9. Sensory Evaluation

Sensory evaluation was conducted with 104 untrained tasters, using an unstructured 9 cm scale for salinity intensity, with the starting point at “completely salt-free” (0) and the endpoint at “excessively salty” (10). Each participant received three samples presented in a randomised manner and coded with random three-digit numbers, according to the methodology used by Beck et al. [10]. The analyses were performed in individual booths with standardised white lighting. The results were obtained by measuring the scales in centimetres (with one decimal place). The sensory study was approved by the Research Ethics Committee of the State University of Maringá (CAAE 18718013.3.0000.0104). In the present study, saltiness intensity was selected as the target attribute because the experimental aim was to test whether a ~30% reduction in sodium could be achieved without loss of salinity perception. Therefore, hedonic and multivariate sensory analyses were not included, as the study did not focus on liking or overall acceptance.

### 2.10. Statistical Analysis

Physicochemical analyses were performed in triplicate as technical replicates obtained from a single extrusion batch for each treatment, representing analytical repeatability rather than independent process variability. Data were subjected to analysis of variance (ANOVA), followed by a comparison of means using Tukey’s test at a 5% significance level, and a correlation analysis. Since each subject evaluated all treatments, sensory data were analysed using a within-subject framework. The residuals did not meet normality assumptions (Shapiro–Wilk, *p* < 0.001); therefore, the nonparametric Friedman test was applied, followed by Wilcoxon paired comparisons with Holm adjustments. Significance level was set at 5%. Statistical analyses were conducted using Sisvar software version 5.6.

## 3. Results

The results presented contribute to the evaluation of replacing common salt in extruded snacks with salt–corn starch composite systems, obtained under two distinct encapsulation conditions: spray drying (SD) and oven drying (OD), both using a salt-to-starch ratio of 70:30 (*w*/*w*).

Table 1 presents the proximate composition, water activity (aw), and sodium content of extruded snacks that were formulated with different salt application treatments. Overall, partial replacement of pure salt with mixtures containing corn starch (SD and OD) did not result in significant changes (*p* > 0.05) in moisture, lipid, protein, and carbohydrate contents, nor in water activity, indicating that modifications in the salting system did not significantly interfere with the overall nutritional matrix of the product.

Moisture content and water activity are fundamental parameters that influence the appearance, juiciness, flavour, texture, and shelf life of extruded products [21]. Moreover, water activity values below 0.6 are associated with greater product stability and a lower risk of microbiological contamination [22]. The absence of statistically significant differences (*p* > 0.05) among the evaluated treatments suggests that incorporating starch into the salt system did not alter the water–matrix interaction after extrusion.

Similarly, lipid, protein, and carbohydrate contents did not show statistically significant differences (*p* > 0.05) among treatments, which can be explained by the fact that the different salts were only applied during the coating step, after extrusion, thus contributing minimally to the product’s total energy and protein fractions.

In contrast, ash and sodium contents showed statistically significant differences (*p* < 0.05), with the highest values observed in the control sample (C) formulated exclusively with pure salt.

Regarding ash content, a significant reduction was observed with salt–corn starch composite systems, with SD and OD values that were statistically similar (*p* > 0.05). This result is consistent, as ash content reflects the inorganic residue remaining after the combustion of organic matter and is directly associated with the food’s mineral content [23]. Thus, diluting salt with corn starch in the SD and OD samples proportionally reduces the mineral load applied to the snack surface, thereby explaining the lower values observed in these treatments compared to the control. Similar results were reported by Amer and Rizk [24] for extruded corn grits snacks without seasoning, which showed an ash content of 1.62%.

Additionally, the sodium content showed a marked and statistically significant reduction in the SD and OD treatments compared to the control, with a decrease of approximately 28%. This sodium reduction was expected, as it corresponds to the decline in salt when starch/salt mixtures in a 70/30 ratio were used in place of 100% salt in the control treatment, indicating no loss of flavour during the flavouring process of the snacks. This reduction is particularly relevant to the nutritional and technological perspectives proposed in this study, as sodium-reduction strategies in processed foods are widely recommended to prevent chronic noncommunicable diseases, such as hypertension and cardiovascular disease [25]. Furthermore, the reduction in sodium content, without significant changes in centesimal composition or water activity, suggests that the salt structure in corn starch is a viable approach to developing extruded snacks with an enhanced nutritional profile without compromising the product’s essential physicochemical characteristics.

Table 2 presents the technological parameters of the extruded snacks, including expansion ratio (ER), specific volume (SV), retraction index (RI), and firmness. Overall, no statistically significant differences (*p* > 0.05) were observed among the evaluated treatments for any of the analysed parameters, indicating that replacing common salt with salt–corn starch composite systems, regardless of the drying method employed, did not compromise the structural and mechanical properties of the extruded snacks.

Expansion is a fundamental characteristic of extruded snacks, as it directly affects consumer acceptability. The physical dimensions and final shape of the snack are primarily determined by the expansion ratio (ER), which also influences chewing quality and visual appeal. The expansion phenomenon is associated with the rheological properties of the dough, especially its viscosity and elasticity, which, in turn, are governed by the proportions of starch, proteins, and fibres present in the formulation. In addition, foods with higher moisture content tend to exhibit lower pressure differentials at the die exit due to the higher melt viscosity, resulting in less expanded products [26,27].

Given the absence of variation among samples in proximate composition parameters (Table 1), it is not surprising that the expansion ratio did not differ significantly among treatments. This result indicates that the salt application method during the coating step did not interfere with the formation of the product’s cellular structure, thereby preserving the characteristic expansion of extruded snacks. Furthermore, the ER values obtained in the present study were higher than those reported by Alefew et al. [28] for rice-based extruded snacks (3.0 ± 0.15) and by Ungureanu-Iuga et al. [29] for corn extrudates (3.70 ± 0.03), demonstrating the adequate technological performance of the developed product.

The retraction index (RI) showed similar values across treatments, indicating dimensional stability after product cooling, further supporting the structural similarity among the samples. Likewise, hardness did not differ statistically among treatments, suggesting that modifying the salting system did not affect the snack’s mechanical resistance. This behaviour is desirable, as firmness is directly associated with the texture and sensory acceptability of extruded snacks and is strongly dependent on the porous structure formed during extrusion.

Table 3 presents the colour parameters of the extruded snacks, expressed as lightness (L*) and the chromatic coordinates a* and b*.

Lightness (L*) showed statistically significant differences (*p* < 0.05) among treatments, with the OD sample showing higher values, indicating a visually lighter product, whereas the control sample (C) exhibited lower lightness. Similar L* results were reported by Uribe-Wandurraga et al. [30] for control corn snacks.

Regarding the b* parameter, associated with the intensity of yellow colouration, statistically significant differences (*p* < 0.05) were also observed, with higher values for the control sample (C) and a progressive reduction in the SD and OD treatments. This behaviour indicates that replacing pure salt with salt–corn starch composite systems reduced the product’s yellowish hue.

The relationship between the L* and b* parameters suggests that treatments with salt–corn starch composite systems, especially OD, resulted in lighter snacks with lower yellow intensity, which may be associated with a more homogeneous distribution of solids on the product’s surface and a lower localised concentration of salt and minerals. In addition, the reduction in b* concomitant with the increase in L* may indicate a lower occurrence of surface browning, contributing to a more uniform and potentially more attractive visual appearance for consumers.

For the a* chromatic coordinate, no statistically significant differences (*p* > 0.05) were observed among treatments, indicating that the characteristic greenish tone of the evaluated snacks was maintained.

Table 4 presents the density and porosity results.

True density (TD) of the salt–starch systems (1.75–1.83 g mL^−1^) fell between that of pure salt (2.16 g mL^−1^) and the literature values reported for starch, which is consistent with the composite nature of the particles [31].

**Figure 1:**
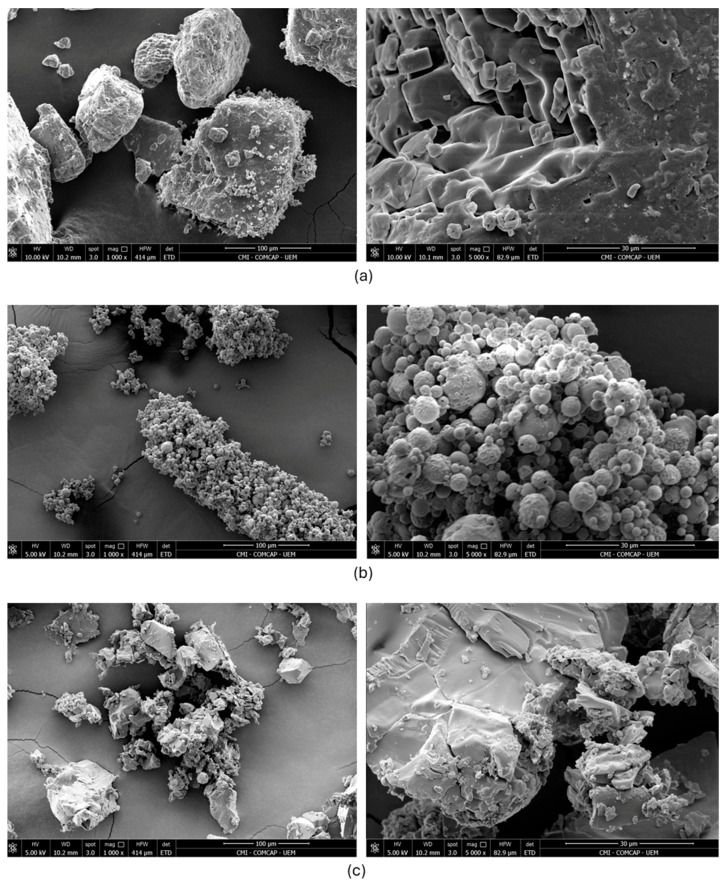
Representative SEM micrographs of (**a**) pure salt (C), (**b**) spray-dried salt–starch particles (SD), and (**c**) oven-dried salt–starch particles (OD).

In contrast, bulk density (LBD/PBD) and the derived porosity (Equation (5)) differed markedly among drying methods. The spray-dried system showed substantially higher porosity than both pure salt and the oven-dried system, whereas OD and C presented comparable porosity values. These results indicate that spray drying promotes the formation of a more void-rich particulate structure under the present processing conditions, which is consistent with the morphological features observed by SEM (Figure 1).

Figure 1 presents representative SEM micrographs of (a) pure salt (C), (b) spray-dried salt–starch particles (SD), and (c) oven-dried salt–starch particles (OD).

SEM micrographs (Figure 1) suggest clear morphological differences among the salt systems. Compared with pure salt and the oven-dried composite, the spray-dried particles exhibit a more open, void-rich architecture, qualitatively consistent with the higher porosity values reported in Table 4. Such porous, sometimes partially hollow morphologies are frequently reported for spray-dried starch-based systems and can increase effective surface area that is available for wetting and dissolution during oral processing [32].

Table 5 presents the tasters’ salinity perceptions across the three treatments.

A significant effect of treatment on perceived saltiness was observed (Friedman: χ^2^(2) = 140.42, *p* < 0.0001). No significant difference was found between C and SD (*p* = 0.56), whereas OD showed lower saltiness than both C (*p* < 0.0001) and SD (*p* < 0.0001). The sensory results showed that the control (C) and spray-drying (SD) samples did not differ significantly in salinity perception by the tasters, indicating that the 28% sodium reduction was not perceived as a difference. On the other hand, when comparing the standard with the sample using oven-dried (OD), a decrease in salinity was observed. This effect aligns with that observed by Noort, Beck, Ming, and Graça [10,11,23,33], who found that the form of salt dispersion contributed significantly to changes in the product’s perceived salinity intensity.

## 4. Discussion

This study demonstrates that spray-dried salt–starch microparticles are an effective strategy for reducing added sodium in extruded snacks while preserving saltiness perception and key quality attributes. Although both salt–starch systems (SD and OD) led to a similar numerical reduction in sodium content (approximately 28%), only the spray-dried treatment maintained perceived saltiness at levels comparable to the control, highlighting the crucial role of particle structure.

Since the spray-dried (SD) and oven-dried (OD) samples had identical salt and starch compositions, the different sensory responses observed between the treatments can be attributed to the drying process itself, not to the simple dilution of sodium. The oven-dried sample, therefore, served as a process control, allowing the isolation of the effects of spray drying on salt release and perception.

Sodium reduction strategies based solely on dilution often compromise flavour intensity, particularly in solid, low-moisture foods such as extruded snacks. In this context, the observed salinity preservation in the SD treatment, along with the density results and micrographs, is consistent with a structure–function interpretation in which the spray-dried process yields a more void-rich particulate architecture (as indicated by SEM and density-derived porosity), potentially favouring earlier brine formation at the tongue surface, thereby supporting saltiness perception despite lower total sodium. These results corroborate previous studies that have consistently shown that salinity perception depends not only on salt concentration but also on the release kinetics and spatial distribution of sodium [10,11,12,23,33]. It is known that rapid dissolution and high local concentrations of sodium on the tongue surface during the initial stages of chewing intensify the perception of salt taste, even when overall sodium levels are reduced.

The absence of a similar sensory effect in the oven-drying treatment, along with higher density values and images showing a more compact particle structure, indicates that the spray-drying-induced structural organisation significantly influenced the perception of salinity in the spray-dried sample. Spray drying commonly yields fine particles with an open and sometimes partially hollow architecture and, consequently, a high effective surface-area-to-volume ratio. In the present study, this structural signature is supported by SEM micrographs and the markedly higher density-derived porosity of the SD system. It is consistent with the literature describing spray-dried porous/hollow salt- or carbohydrate-based systems that may facilitate wetting and promote earlier sodium availability during oral processing [33]. In contrast, oven drying involves starch gelatinisation followed by grinding, resulting in denser, more compact particles that can delay sodium release and reduce the initial salt burst, thereby contributing to the perception of salinity.

It is important to note that the sensory evaluation in this study was intentionally restricted to saltiness intensity, as the primary objective was to determine whether sodium reduction via particle engineering could preserve the product’s perceived salinity. Therefore, hedonic measures, flavour interactions with the herb seasoning, and temporal profiling were not assessed, as they fall outside the scope of the present hypothesis. Future work may incorporate temporal saltiness profiling and consumer acceptance tests to characterise further how engineered salt systems modulate flavour perception beyond the initial salt burst.

Significantly, replacing pure salt with salt–starch microparticles did not affect the proximate composition, water activity, expansion, texture, or dimensional stability of the snacks. These findings indicate that the sodium reduction strategy operated independently of the extrusion process and the product’s structural formation, as the salt systems were applied during the coating step. Maintaining expansion ratio, hardness, and retraction index is particularly relevant, as these parameters strongly influence consumer acceptance and are often negatively affected by formulation changes in extruded products.

Colour changes observed in the SD and OD treatments, characterised by increased lightness and reduced yellow intensity, may be attributed to dilution of the mineral content and to more homogeneous surface coverage by the salt–starch particles. Although statistically significant, these differences are unlikely to compromise consumer acceptance and may even enhance perceptions of product uniformity and cleanliness, depending on market positioning.

From a nutritional and public health perspective, the ability to achieve a nearly one-third reduction in sodium content without perceptible loss of saltiness represents a meaningful advance. Extruded snacks are widely consumed and are recognised contributors to excessive sodium intake, particularly among children and adolescents. Therefore, reformulation strategies that preserve sensory quality are essential to ensure consumer adherence and real-world impact.

This study integrates saltiness perception with complementary physical (density-derived porosity) and morphological (SEM) evidence to support a structure–function rationale consistent with prior reports. Nonetheless, the proposed link between particle architecture and sodium delivery was inferred from these convergent observations rather than being corroborated through dedicated dissolution/ion-release profiling or quantitative surface and size metrics. In addition, sensory testing focused on saltiness intensity and did not address hedonic liking, flavour interactions, or temporal perception. These aspects represent logical next steps to strengthen the mechanistic narrative further and extend consumer-relevant interpretation across product matrices.

Overall, the findings reinforce that sodium reduction should not be approached solely as a compositional challenge but rather as a problem of flavour delivery and perception. Spray-dried salt–starch systems emerge as a promising, scalable, and label-friendly approach aligned with industrial demand for healthier processed foods while maintaining sensory performance.

## 5. Conclusions

This study demonstrates that sodium reduction in extruded corn snacks can be achieved successfully with spray-dried salt–starch microparticles, resulting in a 28% decrease in sodium content while maintaining perceived saltiness and key physicochemical, textural, and visual properties. The effectiveness of spray drying compared to oven drying highlights the importance of particle formation, which positively affects the perception of salinity.

The results indicate that starch-based salt support systems, applied during the coating stage, represent a promising and robust strategy for reformulating snacks, operating without interfering with the product’s structure and composition, except for the salt. From a nutritional standpoint, this approach directly contributes to sodium-reduction targets recommended by public health authorities, offering a practical pathway for industry adoption.

Overall, the results indicate that engineering salt–starch particles by spray drying can preserve saltiness perception at reduced sodium levels and that the SEM morphology together with density-derived porosity provides plausible structural support for a release-based explanation; in practical terms, this porosity-driven modulation of salt delivery represents a promising route for developing extruded snacks with reduced sodium content that is aligned with consumer expectations and health-focused reformulation strategies.

## Figures and Tables

**Table 1:** Average values ^1^ for centesimal composition, water activity (aw), and sodium content in the different treatments of formulated snacks.

Parameters	Treatments ^2^		
	C	SD	OD
Moisture (%)	3.41 ± 0.80 ^a^	3.44 ± 0.27 ^a^	3.01 ± 0.06 ^a^
Lipids (%)	12.36 ± 1.27 ^a^	12.18 ± 0.98 ^a^	11.94 ± 1.17 ^a^
Protein (%)	8.26 ± 0.53 ^a^	8.30 ± 0.46 ^a^	8.07 ± 0.67 ^a^
Carbohydrates (%)	73.51 ± 0.76 ^a^	74.11 ± 0.77 ^a^	74.94 ± 0.55 ^a^
Ash (%)	2.46 ± 0.13 ^b^	1.97 ± 0.07 ^a^	2.04 ± 0.11 ^a^
Wa	0.41 ± 0.03 ^a^	0.40 ± 0.11 ^a^	0.41 ± 0.07 ^a^
Sodium (mg/100 g)	774.2 ± 3.74 ^b^	557.3 ± 6.55 ^a^	571.2 ± 4.96 ^a^

^1^ Different letters following the mean and standard deviation values in the same line indicate significant differences according to the ANOVA test (*p* < 0.05). ^2^ C: control sample containing only pure salt; SD: sample containing spray-dried composite microparticles composed of salt and corn starch (70/30 *w*/*w*); and OD: sample containing a mixture of salt and starch dried in an oven (70/30 *w*/*w*).

**Table 2:** Average values ^1^ of Expansion Ratio (ER), Specific Volume (SV), retraction index (RI), and Hardness in the different treatments of formulated snacks.

Parameters	Treatments ^2^		
	C	SD	OD
ER	4.37 ± 0.28 ^a^	4.21 ± 0.18 ^a^	4.36 ± 0.23 ^a^
SV (mL g^−1^)	14.28 ± 1.49 ^a^	13.98 ± 1.13 ^a^	15.08 ± 0.23 ^a^
RI	0.94 ± 0.05 ^a^	0.93 ± 0.06 ^a^	0.95 ± 0.03 ^a^
Hardness (N)	65.2 ± 3.74 ^a^	68.2 ± 4.04 ^a^	66.3 ± 2.73 ^a^

^1^ Different letters following the mean and standard deviation values in the same line indicate significant differences according to the ANOVA test (*p* < 0.05). ^2^ C: control sample containing only pure salt; SD: sample containing spray-dried composite microparticles composed of salt and corn starch (70/30 *w*/*w*); and OD: sample containing a mixture of salt and starch dried in an oven (70/30 *w*/*w*).

**Table 3:** Average values ^1^ for the colour parameters Luminosity (L*) and chromatic coordinates a* and b* in the different treatments of formulated snacks.

Parameters	Treatments ^2^		
	C	SD	OD
L*	82.28 ± 2.23 ^b^	84.34 ± 1.01 ^ab^	85.05 ± 1.52 ^a^
a*	−7.77 ± 0.35 ^a^	−7.40 ± 0.28 ^a^	−7.28 ± 0.56 ^a^
b*	43.86 ± 1.19 ^b^	41.68 ± 0.99 ^ab^	40.59 ± 1.98 ^b^

^1^ Different letters following the mean and standard deviation values in the same line indicate significant differences according to the ANOVA test (*p* < 0.05). ^2^ C: control sample containing only pure salt; SD: sample containing spray-dried composite microparticles composed of salt and corn starch (70/30 *w*/*w*); and OD: sample containing a mixture of salt and starch dried in an oven (70/30 *w*/*w*).

**Table 4:** Average values ^1^ of loose bulk density (LBD), packed bulk density (PBD), true density (TD), and porosity in the different treatments of formulated snacks.

Parameters	Treatments ^2^		
	C	SD	OD
Packed bulk density (g mL^−1^)	1.27 ± 0.04 ^a^	0.23 ± 0.01 ^c^	1.02 ± 0.02 ^b^
Loose bulk density (g mL^−1^)	1.24 ± 0.03 ^a^	0.21 ± 0.01 ^c^	0.94 ± 0.01 ^b^
True density (g mL^−1^)	2.16 ± 0.07 ^a^	1.83 ± 0.09 ^b^	1.75 ± 0.08 ^b^
Porosity (%)	41.2 ± 0.07 ^b^	87.4 ± 0.07 ^a^	41.7 ± 0.07 ^b^

^1^ Different letters following the mean and standard deviation values in the same line indicate significant differences according to the ANOVA test (*p* < 0.05). ^2^ C: control sample containing only pure salt; SD: sample containing spray-dried composite microparticles composed of salt and corn starch (70/30 *w*/*w*); and OD: sample containing a mixture of salt and starch dried in an oven (70/30 *w*/*w*).

**Table 5:** Average values ^1^ for the analysis of salinity perception in extruded snacks, considering four applications of the formulated treatments.

Treatments ^2^	Mean	Post Hoc Grouping
SD	4.97 ± 0.56	a
C	4.89 ± 0.68	a
OD	3.14 ± 0.77	b

^1^ Different letters indicate significant differences according to paired Wilcoxon tests with Holm correction (*p* < 0.05). Friedman test: χ^2^(2) = 140.42, *p* < 0.0001. ^2^ C: control sample containing only pure salt; SD: sample containing spray-dried composite microparticles composed of salt and corn starch (70/30 *w*/*w*); and OD: sample containing a mixture of salt and starch dried in an oven (70/30 *w*/*w*).

## Data Availability

The original contributions presented in this study are included in the article. Further inquiries can be directed to the corresponding author.
